# Carrier Status for p.Gly61Glu and p.Arg368His *CYP1B1* Mutations Causing Primary Congenital Glaucoma in Iran

**DOI:** 10.18502/jovr.v16i4.9747

**Published:** 2021-10-25

**Authors:** Ali Heshmati, Peyman Taghizadeh, Hamid Ahmadieh, Mehdi Yaseri, Fatemeh Suri, Mahsa Alizadeh, Marjan Dadashzadeh, Hajar Khatami, Monireh Moradkhah Navi, Parisa Zamanparvar, Hassan Behboudi, Elahe Elahi

**Affiliations:** ^1^School of Biology, University College of Science, University of Tehran, Tehran, Iran; ^2^Ophthalmic Research Center, Research Institute for Ophthalmology and Vision Science, Shahid Beheshti University of Medical Sciences, Tehran, Iran; ^3^Department of Epidemiology and Biostatistics, School of Public Health, Tehran University of Medical Sciences, Tehran, Iran; ^4^Astaneh Health Center, Astaneh, Guilan, Iran; ^5^Rasht Health Center, Rasht, Guilan, Iran; ^6^Anzali Health Center, Anzali, Guilan, Iran; ^7^Talesh Medical Center, Talesh, Guilan, Iran; ^8^Lahijan Medical Center, Lahijan, Guilan, Iran; ^9^Department of Ophthalmology, Guilan University of Medical Sciences, Rasht, Iran

**Keywords:** CYP1B1, Guilan, Iran, p.Arg368His, p.Gly61Glu, Primary Congenital Glaucoma

## Abstract

**Purpose:**

To estimate carrier frequencies of *CYP1B1 *mutations p.Gly61Glu and p.Arg368His, respectively, in Talesh and the east of Guilan province in Iran with a maximum error of 2%. Previously, it was shown that these *CYP1B1 *mutations may be relatively prevalent in these regions.

**Methods:**

Population-based screenings were performed. DNA was extracted from saliva samples of 1036 individuals from Talesh and 3029 individuals from the east of Guilan. P.Gly61Glu and p.Arg368His screenings were performed, respectively, by RFLP and ARMS-based PCR protocols. For confirmation, the DNA of individuals with mutations was sequenced using the Sanger protocol.

**Results:**

Nine individuals from Talesh (0.86%; 95%CI: 0.45–1.64%) carried the p.Gly61Glu mutation, and 73 from the east of Guilan (2.41%; 95%CI: 1.91–3.04%) carried p.Arg368His. There was no significant difference in frequencies between urban and rural regions of the various cities, nor among four cities within the east of Guilan.

**Conclusion:**

The frequencies of p.Gly61Glu carriers in Talesh and of p.Arg368His carriers in the east of Guilan were within the 95% confidence interval of a previous study based on screenings of fewer individuals. The reliability of the recent estimates is higher, as the confidence interval for p.Gly61Glu decreased from 6.5% to 1.19% and the interval for p.Arg368His decreased from 4% to 1.13%. Based on the new findings, the maximum expected frequency of p.Gly61Glu carriers in Talesh is 1.64%, and of p.Arg368His carriers in the east of Guilan is 3%. The need for performing premarital screenings in the respective cities can be evaluated.

##  INTRODUCTION

Glaucoma is a major cause of irreversible blindness worldwide.^[1]^ On the basis of the anatomy of the anterior chamber drainage angle and age of onset, primary glaucoma is classified as primary congenital glaucoma (PCG; OMIM 231300), primary open angle glaucoma (POAG), and primary angle closure glaucoma (PACG). PCG which is the subject of this report is the most severe form of glaucoma.^[2]^ It is characterized by an anatomical defect (trabeculodysgenesis) in the trabecular meshwork and the age of onset in the neonatal period or before the age of three years.^[3]^ PCG occurs in both sporadic and familial patterns. In familial cases, inheritance is usually autosomal recessive.^[3,4]^ While less common than the adult onset forms, PCG is an important cause of childhood blindness.^[5,6]^ The incidence of PCG is geographically and ethnically variable, and highest in populations with high rates of consanguineous marriages such as Saudi Arabia (1:2500).^[7,8]^

Genetic analysis of recessive PCG-affected families have identified four associated loci, GLC3A – D.^[9,10]^ The causative gene in two of the loci have been identified: *CYP1B1* in GLC3A and *LTPBP2* in GLC3D that encode, respectively, cytochrome P450 family 1 subfamily B polypeptide 1 and latent transforming growth factor-β binding protein 2. More recently, mutations in *TEK* that encodes tunica interna endothelial cell kinase have been reported in both sporadic and autosomal dominant familial PCG patients.^[4,11]^ Mutations in *CYP1B1* are by far the most commonly known cause of PCG. Nevertheless, the proportion of PCG patients whose disease is attributable to *CYP1B1* is different among populations, ranging from 20% in Japan to 90% in Saudi Arabia and 100% in Slovakia Roma.^[7,12,13,14]^ More than 130 putative PCG-causing mutations distributed in the coding regions of *CYP1B1* have been documented (Human Genome Mutation Database; http://www.hgmd.cf.ac.uk/ac/index.php). The degree of heterogeneity of *CYP1B1 *mutations in different populations is quite variable.^[15]^

A study in which *CYP1B1* was screened in 104 Iranian PCG patients identified a mutation in approximately 70% of the patients.^[16]^ This suggested that mutations in *CYP1B1* significantly contributes to PCG burden in Iran. Although 19 different disease-associated mutations were identified, four of these constituted 77.3% of the identified mutated alleles. The four common mutations caused p.Gly61Glu, p.Arg368His, p.Arg390His, and p.Arg469Trp. Another important finding of the same study was that PCG incidence is not evenly distributed in Iran, and that incidence is relatively high in the west and northwest of Iran. Guilan, which is located in the northwest of Iran, was one of the provinces with a high incidence of PCG.

The findings of the early study summarized above suggested that the frequency of PCG-unaffected individuals in Iran who harbor one allele of the four aforementioned *CYP1B1* mutations may be considerable, particularly in regions with high incidence of the disease. It was considered that a correspondingly relatively high frequency of marriages may occur between carriers of these mutations. The likelihood of giving birth to a PCG-affected offspring would increase in such marriages. In this light, a pilot study was performed in which the four common *CYP1B1* mutations were screened in 700 individuals from the province of Guilan.^[17]^ The individuals screened were from a cross-sectional population-based survey in Guilan that included clusters in urban and rural areas.^[18]^ Only carriers of the p.Gly61Glu and p.Arg368His–causing mutations were identified in the more recent survey, which suggested that regional frequencies of *CYP1B1* mutations do not necessarily mirror national frequencies. Furthermore, the p.Gly61Glu and p.Arg368His-causing mutations were not geographically randomly distributed in Guilan; most of the individuals with the p.Gly61Glu mutation were from Talesh, and most of the individuals with the p.Arg368His mutation were from the east of Guilan. The study whose results are reported here was performed to gain a more accurate assessment of the frequency of the c.182G > A mutation that causes p.Gly61Glu in the population of Talesh and of the c.1103G > A mutation that causes p.Arg368His in the population of cities in the east of Guilan. These objectives were achieved by screening larger numbers of individuals.

##  METHODS

This research was performed in accordance with the Declaration of Helsinki and with the approval of the ethics board of the University of Tehran and the Ophthalmic Research Center of Shahid Beheshti University of Medical Sciences.

### Population Sampling

We aimed to perform population-based mutation screenings to achieve estimates of the frequency of p.Gly61Glu *CYP1B1* mutation carriers in the population of Talesh and of p.Arg368His carriers in the population of the east of Guilan with a maximum error of 2%. The frequency of p.Gly61Glu carriers in Talesh based on screening of 173 individuals in an earlier study was 2.9% (95% confidence interval: 0.8–7.3%).^[17]^ To achieve a maximum error of 2%, it was calculated that 1000 individuals would need to be screened in the new survey. We chose to recruit 1400 individuals (485 males, 915 females) as a safety net to assure successful screening in at least 1000 individuals. The frequency of p.Arg368His carriers in the east of Guilan based on the screening of 268 individuals was 2.2% (95% confidence interval: 0.8–4.8%.^[17]^ To achieve a maximum error of 2%, it was calculated that 3012 individuals would need to be screened in the new survey.^[19]^ We choose to recruit 3400 individuals (1088 males, 2312 females). The east of Guilan is constituted by the urban and rural regions of the cities Rasht, Lahijan, Bandar Anzali, and Astaneh Ashrafieh.

The size of the population of the cities studied and their urban/rural distribution were obtained from the Statistical Center of Iran (amar.org.ir). The number of individuals studied from each of the cities and the urban/rural distribution of each of these are shown in Tables 1 and 2. The urban/rural distribution of individuals recruited was always proportional to the urban/rural distribution of the population of each of the cities in these two regions. The distribution of the 3400 individuals recruited from east Guilan was proportional to the population size of the four cities that constitute east of Guilan. Individuals from each rural or urban region were recruited according to a cluster sampling protocol.^[20]^ For this purpose, homes in residential areas were randomly selected based on postal codes obtained from Iran Post which is the national postal service of Iran. Sample collection was done at selected residences. Only one individual from any single home was recruited.

### *CYP1B1* Mutation Screening 

Each participating individual after having thoroughly rinsed her/his mouth with water provided 1–2 ml saliva. The 15 ml tubes containing the saliva samples were retained at room temperature at a medical center in Guilan until delivery to the University of Tehran. The samples were transported in batches of 40 over a period of approximately two months. For DNA extraction, 1.5 cc saliva was added to a 15 ml tube that contained 1.5 cc lysis buffer (13 mM EDTA, 11 mM sodium citrate, 10 mM Tris-HCl pH 8, 1% SDS). The protocol for DNA extraction was immediately pursued, or the samples were stored in the lysis buffer at 4°C for up to one week or at –20°C for up to 10 months. For samples delivered to the University of Tehran within a week of donation, the tubes containing the samples and lysis buffer were vortexed at high speed for approximately 1 min to promote cell and nucleus lysis. For samples with a longer lapse between donation and delivery, the contents of the tubes were mixed but vortexing was not necessary. Subsequently, 400 μl of each tube was transferred to an Eppendorf tube, and 150 μl 6 M NaCl and 600 μl chloroform were added. The tubes were inverted 10 times, retained at room temperature for 5 min, and then centrifuged in a microfuge for 15 min at 14000 RPM. The clear supernatant was transferred to a new tube and an equal volume of cold (–20°C) isopropanol was added. After several times of inversion, clots of precipitated DNA were often visible. The tubes were placed at –20°C for 30 min to overnight, and subsequently centrifuged for 10 min at 12000 RPM at 4°C. After centrifugation, the supernatant was discarded, and the precipitated DNA was washed with 70% cold ethanol, dried, and dissolved in 30 μl H ${}_{2}$ O. The concentration of DNA in the samples was quite variable. Appropriate dilutions were made, and 20 ng DNA was used as template in subsequent PCR reactions.

**Figure 1:**
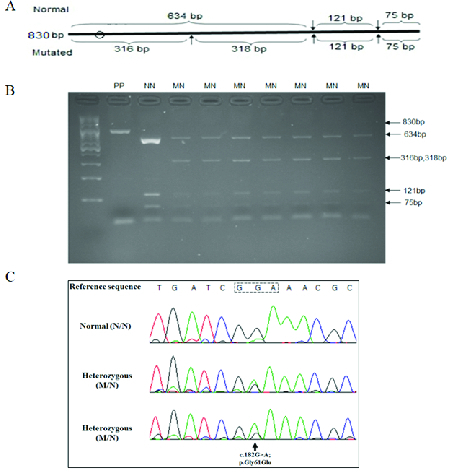
P.Gly61Glu mutation in *CYP1B1* caused by the c.182G > A mutation in exon 2 of the encoding gene. (A) Schematic diagram of 830 bp PCR amplicon that includes part of exon 2 of *CYP1B1*. *Taq*1 restriction enzyme recognition sites in the amplicon containing normal and mutated exon 2 sequences, respectively, are shown by arrows above and below the amplicon. The size of DNA digestion products predicted for the two types of alleles are indicated. The circle shows position of the start of exon 2 within the PCR amplicon. (B) PCR products relevant to screening of p.Gly61Glu. PP, the exon 2 containing PCR amplicon without restriction enzyme treatment; NN, digestion pattern of the amplicon of an individual known to be homozygous for normal sequence; MN, electrophoresis patterns of digestion products of seven of the nine individuals from Talesh who were carriers of the p.Gly61Glu mutation. (C) DNA sequence chromatograms of a wild type *CYP1B1* genotype (top) and the heterozygous genotype for the two c.182G > A mutation carriers that causes p.Gly61Glu (bottom).

The p.Gly61Glu causative mutation was screened in the DNA of the individuals from Talesh by a restriction fragment length polymorphism (RFLP) protocol, and the p.Arg368His causative mutation in the DNA of the individuals from the cities of the east of Guilan was screened by an amplification-refractory mutation system (ARMS) protocol as previously described.^[17]^ Briefly, an 830 bp exon 2 fragment that includes c.182G > A that causes p.Gly61Glu, and an 872 bp exon 3 fragment that includes c. 1103G > A that causes p.Arg368His were PCR amplified. The restriction enzymes *Taq*I was used in the RFLP reactions for detection of c.182G > A. The unmutated exon 2 amplicon contains two *Taq*I recognition sites. The c.182G > A mutation creates a novel recognition site, and digestion by *Taq*I results in a changed pattern upon electrophoresis of digested products [Figure 1A]. For the ARMS reaction pertaining to c.1103G > A that causes p.Arg368His, wild type-specific and mutation-specific PCR primers were designed such that amplification of wild type alleles would proceed only in the presence of the primer specific for the unmutated sequence, and amplification of mutated alleles would proceed only in the presence of the primer specific for the mutated sequence. Primers for amplification of a control irrelevant DNA fragment were included in all ARMS reactions. The sequences of all primers used have been published.^[17]^ For confirmation of putative mutant alleles identified, these alleles were sequenced using the Sanger dideoxynucleotide termination protocol.

**Figure 2:**
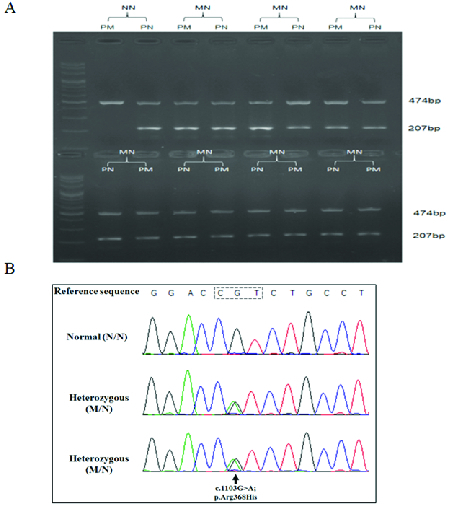
P.Arg368His mutation in *CYP1B1* caused by the c.1103G > A mutation in exon 3 of the encoding gene. (A) Electrophoresis patterns of PCR products relevant to screening of p.Arg368His by ARMS protocol. Two patterns are presented for each individual, one that represents products obtained with primer specific for normal allele (PN) and another that represents products obtained with primer specific for mutated allele (PM). The 474 bp fragment is the control fragment. The 207 bp fragment is from *CYP1B1*. NN, electrophoresis pattern of PCR products of individual known to be homozygous for the normal allele; MN, electrophoresis patterns of seven of representative individuals from east of Guilan who were carriers of the p.Arg368His mutation. (B) DNA sequence chromatograms of a wild type *CYP1B1* genotype (top) and two heterozygous genotypes for the c.1103G > A mutation that causes p.Arg368His (bottom).

### Statistical Analysis

To present the prevalence of the mutations, the 95% confidence interval was derived using exact binomial method. The cluster effect was considered by incorporating the design effect into the calculations. All statistical analyses were performed using STATA 14.0 (STATA Corp., Texas, USA).

##  RESULTS

Successful genotyping results were achieved for the DNA of 1036 of the 1400 individuals (74.0%) recruited from Talesh. Based on electrophoretic patterns, nine individuals (four male, five female) were carriers of the p.Gly61Glu mutation, corresponding to a carrier frequency of 0.86% (95% confidence interval: 0.45–1.64%) [Figure 1B; Table 1]. Sanger sequencing confirmed the electrophoresis results in all the nine individuals [Figure 1C]. The carrier frequencies of the Talesh urban (0.85%) and rural regions (0.88%) were not significantly different.

Successful genotyping results were achieved for the DNA of 3029 of the 3400 individuals (89.1%) recruited from the cities of the east of Guilan. Based on electrophoretic patterns, 73 individuals (12 male, 61 female) were carriers of the p.Arg368His mutation, corresponding to a carrier frequency of 2.41% (95% confidence interval: 1.91–3.04%) [Figure 2A; Table 2]. Sanger sequencing confirmed the electrophoresis results in 20 randomly chosen putative mutation carriers [Figure 2B]. The carrier frequencies of the four cities of the east of Guilan were not significantly different. Similarly, the carrier frequencies of the urban and rural regions in each of the cities were not significantly different [Table 2].

##  DISCUSSION

Whereas earlier genetic screenings pertaining to glaucoma were performed using DNA extracted from the peripheral blood, DNA extracted from cells in the saliva was used in the present study.^[15,17]^ Clearly, obtaining saliva samples for population-based studies and for studies with a relatively large number of participants is more feasible than getting blood samples. Whereas the quality of blood-derived DNA is almost invariably suitable for genetic and molecular studies, this is not necessarily the case for saliva-derived DNA. In our hands, approximately 75% of the DNAs of the Talesh samples and 90% of the DNAs of the samples from the cities of east Guilan functioned as template in PCR reactions. We believe that lack of success in genotyping for some samples was primarily due to presence of debris in the samples that could have been eliminated by better washing of the mouth prior to saliva donation. Issues of delay in transport contributed to a lesser extent.

Our study design allowed comparison of the frequency of mutation carriers in urban and rural regions of each of the cities studied. As PCG inheritance is usually autosomal recessive, it was considered that PCG frequencies and correspondingly mutation carrier frequencies may be higher in rural regions because of presumed higher rates of consanguineous marriages. In fact, statistically significant differences between frequencies of mutation carriers in urban and rural regions of the various cities was not observed. The apparent relatively high frequency of carriers in the rural region of Bandar Anzali (5.66%) may be due to sample size issues.

The most important findings of the present study are estimates of p.Gly61Glu and p.Arg368His mutation carrier frequencies, respectively, in Talesh and the cities of the east of Guilan. The estimated frequency of p.Gly61Glu carriers in Talesh based on screening of 1036 individuals was 0.86% which is notably less than the earlier estimate of 2.9% based on screening of 173 individual. The frequency estimated in the present study is at the lower edge of the earlier 95% confidence interval frequency range (0.8–7.3%). As expected, the range of the 95% confidence interval (1.64–0.45% = 1.19%) is approximately five times less than the range of the 95% confidence interval (0.8–7.3% = 6.5%) of the earlier screening. This clearly suggests that the recent finding is more informative, and that the maximum expected frequency of p.Gly61Glu carriers in Talesh is 1.64% rather than 7.3%.

The estimated frequency of p.Arg368His carriers in the east of Guilan based on screening of 3029 individuals was 2.41% which is close to the earlier estimate of 2.2% based on screening of 268 individual. The range of the 95% confidence interval (3.04–1.91% = 1.13%) is approximately four times less than the range of the 95% confidence interval (4.8–0.8% = 4%) of the earlier screening. Again, the recent finding is more reliable, and suggests that the minimum estimated frequency of p.Arg368His carriers in the east of Guilan is 1.91% rather than the previous estimate of 0.8%. The carrier frequency may be as high as 3%.

The new and more reliable estimates of carrier frequencies as compared to the estimates of the earlier study are encouraging in the sense that the maximum frequency of p.Gly61Glu mutation carriers in Talesh has decreased from 7.3% to 1.64%, and the maximum frequency of p.Arg368His carriers in the east of Guilan has decreased from 4.8% to 3.04%. The possibility that the frequency of p.Arg368His may be higher in some localities within the east of Guilan such as Astaneh Ashrafieh can be assessed by larger screenings within these localities [Table 2]. The need for screening of the p.Gly61Glu and p.Arg368His mutations before marriage in the respective regions should be considered. The appropriateness of implementation of measures to encourage screening of specific genes or specific mutations before marriage depends on various factors including cost and feasibility of screening and cost and size of disease burden. Based on the 95% confidence level (0.45–1.64%) for frequency of p.Gly61Glu carriers in Talesh, and assuming random mating, it is expected that between 0.002% (0.45% × 0.45%) and 0.003% (1.64% × 1.64%) of marriages in Talesh will be carriers of this mutation. One fourth of the offspring of such marriages are expected to be affected with PCG. Therefore, assuming equal numerical contribution of all marriages to the population of the following generation, it is expected that among one million children born in Talesh, 5–7.5 ([0.25 × 0.002%] – [0.25 × 0.003%]) will be affected with PCG as a consequence of marriages between p.Gly61Glu mutation carriers. The figures for the p.Arg368His mutation in the east of Guilan are somewhat more disturbing. Assuming random mating, between 0.036% (1.91% × 1.91%) and 0.103% (3.04% × 3.04%) of marriages in this region of Guilan will be between carriers of the p.Arg368His mutation. It can be expected that among 100,000 children born in the eastern region of Guilan, 9–25.8 ([0.25 × 0.036%] – [0.25 × 0.103]) will be affected with PCG as a consequence of marriages between p. Arg368His mutation carriers. It is to be noted that the ARMS protocols developed for screening of the p.Arg368His-causing mutation is relatively inexpensive and easy to implement in clinical laboratories. It is also to be noted that premarital mutation screening programs in Iran and elsewhere for thalassemia and cystic fibrosis have resulted in reduced disease incidence.^[21,23,24]^ With respect to PCG, to the best of our knowledge, premarital screenings is not routinely performed in any country. This protocol is perhaps best advisable and feasible in a country such as Saudi Arabia where the frequency of the disease is relatively high and where a single *CYP1B1* mutation makes a very significant contribution to disease incidence.^[7,8]^

##  Financial Support and Sponsorship

The research was funded and sponsored by the Vice Chancellor for Research and Technology of Guilan University of Medical Science, the Ophthalmic Research Center of Shahid Beheshti University of Medical Sciences, and the Research Division of the University of Tehran.

##  Conflicts of Interest

There are no conflicts of interest.
