# Erratum: Carrier Status for p.Gly61Glu and p.Arg368His *CYP1B1* Mutations Causing Primary Congenital Glaucoma in Iran

**DOI:** 10.18502/jovr.v17i2.10809

**Published:** 2022-04-29

**Authors:** Ali Heshmati, Peyman Taghizadeh, Hamid Ahmadieh, Mehdi Yaseri, Fatemeh Suri, Mahsa Alizadeh, Marjan Dadashzadeh, Hajar Khatami, Monireh Moradkhah Navi, Parisa Zamanparvar, Hassan Behboudi, Elahe Elahi

The authors have informed the journal that Tables 1 and 2 cited in the original article were missing in the full-text PDF and HTML of the article published in the *Journal of Ophthalmic and Vision Research *Volume 16 Issue 4. The online version of the article has therefore been updated on April 29, 2022 and can be accessed from https://doi.org/10.18502/jovr.v16i4.9747.

**Table 1:** Screening of p.Gly61Glu in the city of Talesh in Guilan.


	**No. individuals screened**	**No. mutation carriers**	Frequency of carriers*	**95% Confidence interval of mutation carriers**
Total	1036	9	0.86%	0.45% - 1.64%
Urban	584	5	0.85%	0.37% - 1.99%
Rural	452	4	0.88%	0.34% - 2.25%
	
	

**Table 2:** Screening of p.Arg368His in cities of the east of Guilan province.


	**No. individuals screened**	**No. mutation carriers**	Frequency of carriers*	**95% Confidence interval of mutation carriers**
All of east of	Total	3029	73	2.41%	1.91% - 3.04%
Gilan	Urban	2160	51	2.36%	1.08% - 3.09%
	Rural	869	22	2.53%	1.68% - 3.8%
Bandar Anzali	Total	389	11	2.82%	1.57% - 4.99%
	Urban	336	8	2.38%	1.21% - 4.63%
	Rural	53	3	5.66%	1.94% - 15.37%
Lahijan	Total	376	5	1.32%	0.58% - 3.15%
	Urban	226	4	1.77%	0.69% - 4.46%
	Rural	150	1	0.67%	0.12% - 3.68%
Rasht	Total	2095	54	2.58%	1.98% - 3.35%
	Urban	1481	37	2.50%	1.82% - 3.42%
	Rural	614	17	2.77%	1.74% - 4.39%
Astaneh Ashrafieh	Total	169	3	1.78%	0.61% - 5.09%
	Urban	117	2	1.71%	0.47% - 6.02%
	Rural	52	1	1.92%	0.34% - 10.12%
	
	

